# Effectiveness of Problem-Based Learning (PBL) in specialist physician training for kidney transplantation

**DOI:** 10.1097/MD.0000000000047684

**Published:** 2026-02-20

**Authors:** Chen Gao, Jiyuan Li, Kankan Shui, Xubiao Xie, Longkai Peng

**Affiliations:** a Department of Kidney Transplantation, The Second Xiangya Hospital of Central South University, Changsha City, Hunan Province, China

**Keywords:** clinical reasoning, kidney transplantation, medical education, objective structured clinical examination, problem-based learning, specialist physician training

## Abstract

This retrospective study evaluated the effectiveness of a Problem-Based Learning (PBL) approach in specialist physician training for kidney transplantation. Trainees enrolled between January and December 2024 were divided to either a traditional teaching cohort or a PBL-based instructional cohort. The PBL curriculum incorporated case-based inquiry, structured group discussions, evidence retrieval, and multidisciplinary clinical integration, while the control group received lecture-centered instruction. Educational outcomes were assessed across theoretical knowledge, clinical reasoning, multidisciplinary case discussion performance, Objective Structured Clinical Examination (OSCE) results, and trainee satisfaction. A total of 115 trainees were included, with comparable baseline demographic and training characteristics between groups. The PBL group demonstrated significantly higher theoretical examination scores across all transplant-related domains, superior clinical reasoning performance, and higher proficiency in multidisciplinary discussion assessments. OSCE scores were also significantly higher in the PBL cohort, reflecting enhanced applied clinical skills in postoperative monitoring, immunosuppressive drug interpretation, recognition of acute rejection, and transplant counseling. Additionally, trainee satisfaction was markedly greater in the PBL group, indicating improved perceived relevance and learning experience. These findings suggest that PBL provides a more effective and comprehensive framework for developing core competencies required in kidney transplantation training compared with traditional instructional methods.

## 1. Introduction

Kidney transplantation remains the preferred renal replacement therapy for patients with end-stage kidney disease, offering superior long-term survival, improved quality of life, and reduced healthcare utilization compared with maintenance dialysis. As transplantation programs continue to expand worldwide, the clinical management of transplant recipients has become increasingly complex, requiring physicians to integrate advanced knowledge of transplant immunology, perioperative medicine, immunosuppressive pharmacotherapy, and long-term surveillance for complications such as acute rejection, opportunistic infections, and chronic allograft dysfunction.^[1-3]^ The multidisciplinary nature of transplant care demands a high-level of clinical reasoning, procedural proficiency, and interprofessional communication. Therefore, the cultivation of competent transplant physicians is an essential component of optimizing patient outcomes and ensuring the sustainability of transplantation services.

Traditional instructor-centered training models, characterized by didactic lectures and passive knowledge acquisition, have been the dominant approach in postgraduate medical education. Although effective for disseminating theoretical concepts, traditional methods may not adequately promote the higher-order cognitive processes needed to manage complex transplantation scenarios. Kidney transplantation often involves rapidly evolving clinical conditions, intricate diagnostic pathways, and individualized immunosuppressive strategies. Under such circumstances, the ability to identify clinical priorities, synthesize multidisciplinary information, and make timely evidence-based decisions is essential.^[4,5]^ Educational models that fail to engage trainees in active problem-solving may limit the development of these competencies. Problem-Based Learning (PBL) offers an alternative instructional strategy that emphasizes learner-centered inquiry, self-directed learning, and collaborative clinical reasoning. By organizing instruction around realistic clinical cases, PBL encourages trainees to define learning objectives, integrate knowledge across domains, evaluate emerging evidence, and apply theoretical principles to practice.^[6,7]^ Prior studies in undergraduate and general medical education have shown that PBL can improve critical thinking, diagnostic accuracy, and long-term retention of knowledge. However, its application within highly specialized fields – particularly in kidney transplantation, where clinical decision-making is complex and time-sensitive has not been extensively characterized.

Given the complexity of transplant patient management and the growing emphasis on competency-based medical education, there is a need to delineate whether PBL-based training can enhance the essential competencies required of kidney transplantation specialists. An educational approach that enhances theoretical knowledge, clinical reasoning, procedural skills, and interdisciplinary communication may offer significant value in preparing physicians for the multifaceted challenges inherent to transplant care. In this context, evaluating the impact of a structured PBL curriculum within specialist training holds important implications for the advancement of postgraduate transplant education and the broader improvement of clinical practice.

## 2. Materials and methods

### 2.1. Study design

This study was approved by the Ethics Committee of The Second Xiangya Hospital of Central South University. This study was designed as a retrospective cohort study based on routinely collected educational assessment data from a standardized specialist physician training program in kidney transplantation conducted at our institution between January and December 2024.

Trainees were categorized into 2 cohorts according to the instructional model implemented during their training period, rather than being prospectively assigned by the investigators. During the study period, the training program transitioned from a traditional lecture-based curriculum to a structured PBL curriculum as part of routine educational reform. Trainees who completed the program before the implementation of PBL constituted the traditional teaching cohort, while those trained after implementation formed the PBL cohort. Group allocation was therefore determined by institutional curriculum scheduling, not by trainee selection, instructor preference, or investigator intervention. The study adhered to the ethical principles outlined in the Declaration of Helsinki and received approval from the institutional medical ethics committee. Written informed consent was obtained from all participating trainees prior to data collection.

### 2.2. Control group: traditional teaching protocol

Trainees in the control group received a conventional instructor-centered teaching protocol. The curriculum consisted primarily of didactic lectures delivered by senior transplant physicians, supplemented by textbook-based self-study. Core content included renal transplantation indications and contraindications, donor–recipient evaluation, surgical procedures, postoperative immunosuppressive regimens, and management of common complications such as delayed graft function, acute rejection, opportunistic infections, and metabolic disorders. Clinical exposure was arranged through fixed rotations in transplant surgery, nephrology, and perioperative care units. Teaching sessions emphasized theoretical knowledge acquisition, and trainee participation was mainly passive. Clinical case discussions followed a teacher-led format, with limited opportunities for independent inquiry or problem-solving. Assessment methods included written examinations and periodic competency evaluations conducted according to institutional standards.

### 2.3. Observation group: PBL-based training protocol

Trainees in the observation group participated in a structured PBL protocol designed to support the acquisition of clinical knowledge and decision-making skills related to kidney transplantation. The PBL curriculum was delivered in small groups of 6 to 8 trainees and facilitated by transplant physicians who had received standardized PBL tutor training. Teaching activities were scheduled on a weekly basis and followed a uniform instructional framework. Each teaching module began with a clinical scenario developed from common or representative problems in kidney transplantation practice, including donor and recipient evaluation, perioperative graft management, immunosuppressive therapy adjustment, diagnosis and classification of acute rejection, management of delayed graft function, and identification of infectious or metabolic complications. Trainees reviewed the scenario, identified key clinical problems, and formulated learning objectives. Independent evidence retrieval was required, and trainees were instructed to consult clinical guidelines, high-quality reviews, and relevant primary literature.

During the PBL sessions, trainees presented their findings, discussed diagnostic and therapeutic strategies, and evaluated the supporting evidence. Tutors guided the discussion process, ensured completeness of the problem analysis, and promoted structured reasoning without providing direct answers. Emphasis was placed on integrating surgical, nephrological, immunological, and infectious disease considerations in the clinical reasoning process. The PBL protocol incorporated additional activities to reinforce applied clinical skills. These included case-based discussions during multidisciplinary transplant ward rounds, participation in postoperative management meetings, review of transplant pathology findings, and simulation-based exercises for perioperative complication management. Trainees were required to complete written case analyses after each module to summarize key clinical decisions and rationale.

### 2.4. Assessment criteria

Assessment outcomes were defined according to the core objectives of specialist physician training in kidney transplantation and were evaluated as follows:

1. Baseline characteristics: age, sex, postgraduate year (PGY-1/2/3), duration of prior nephrology training, duration of prior kidney transplantation rotation, and baseline written examination scores (0–100) were used to describe and compare the initial status of trainees in the 2 groups.
2. Theoretical knowledge: post-training theoretical knowledge was assessed using a standardized written examination (0–100). The exam covered 5 domains: donor and recipient evaluation, perioperative management, immunologic risk assessment, immunosuppressive drug monitoring, and early/late postoperative complications. Each domain was scored on a 0 to 20 subscale. An overall score ≥ 80 points was defined as high theoretical performance.
3. Clinical reasoning ability: clinical reasoning was evaluated using structured case analyses of kidney transplantation scenarios. Performance was scored in 4 domains – problem identification, diagnostic reasoning, management strategy formulation, and evidence justification – each on a 0 to 25 scale. The sum yielded a total clinical reasoning score (0–100). An overall score ≥ 80 points was considered high clinical reasoning performance.
4. Multidisciplinary case discussion performance: performance in multidisciplinary kidney transplantation case discussions was scored by faculty in 4 domains: accuracy of clinical interpretation, completeness of problem identification, clarity of reasoning and presentation, and integration of multidisciplinary information (surgery, nephrology, immunology, infectious diseases). Each domain was scored 0 to 25, producing an overall discussion score (0–100). Scores ≥ 80 points were categorized as high discussion performance.
5. Objective Structured Clinical Examination (OSCE): operational and applied clinical skills were assessed using an OSCE comprising stations on postoperative monitoring, interpretation of immunosuppressant drug levels, recognition of acute rejection signs, and communication in transplant counseling. Each station was scored 0 to 25 using predefined checklists, and the overall OSCE score was calculated on a 0 to 100 scale. An overall OSCE score ≥ 80 points was defined as satisfactory performance.
6. Trainee satisfaction: trainee satisfaction with the teaching protocol was measured using a structured questionnaire based on a 5-point Likert scale (1 = very dissatisfied, 5 = very satisfied). Items included satisfaction with teaching content, teaching format, perceived relevance to clinical practice, overall learning experience, and an overall satisfaction score. An overall score ≥ 4 points was defined as satisfactory satisfaction.

### 2.5. Data collection

All data were collected after completion of the training cycle for both the control (traditional teaching) group and the PBL group. Baseline demographic and training-related variables (age, sex, PGY level, prior nephrology training duration, and prior kidney transplantation rotation duration) were obtained from the institutional training management system by designated administrative staff and verified by the research team. Baseline and post-training written examination scores, clinical reasoning assessments, multidisciplinary discussion evaluations, and OSCE results were collected from routinely administered teaching assessments. Examinations and OSCEs were organized by the teaching office according to standardized procedures, and scoring was performed using predefined answer keys and checklists. The clinical reasoning and multidisciplinary discussion scores were recorded on standardized scoring sheets by trained faculty members who were familiar with the assessment rubrics. Satisfaction questionnaires were distributed to all trainees at the end of the training period, completed anonymously, and collected on the same day. All paper-based scores and questionnaires were entered into an electronic database by 2 independent researchers using double data entry to reduce transcription errors. Any discrepancies between entries were resolved by checking the original records. Only trainees with complete baseline and post-training assessment data were included in the analysis, consistent with the reported sample of 115 trainees. All data were de-identified before analysis to protect participant confidentiality.

### 2.6. Statistical analysis

All statistical analyses were conducted using IBM SPSS Statistics version 26.0 (IBM Corp., Armonk). Continuous variables were examined for normality using the Shapiro–Wilk test. Normally distributed variables were expressed as mean ± standard deviation and compared between groups using independent-samples *t*-tests with Welch’s correction when appropriate. Categorical variables were presented as frequencies and percentages and analyzed using the χ^2^ test. All statistical analyses were 2-sided, and a *P* value < .05 was considered statistically significant.

## 3. Results

### 3.1. Baseline characteristics

A total of 115 trainees were included in the study, with 56 in the control group and 59 in the PBL group. There was no significant difference in age between the 2 groups (30.9 ± 2.8 vs 31.4 ± 2.4 years; *t* = −1.026, *P* = .305). The proportion of male trainees was comparable (53.6% vs 55.9%; χ^2^ = 0.065, *P* = .799). The distribution of training level (PGY-1/2/3) did not differ significantly between the control and PBL groups (37.5%/35.7%/26.8% vs 35.6%/27.1%/37.3%; χ^2^ = 1.692, *P* = .429). Prior nephrology training duration was similar in the 2 groups (8.8 ± 3.0 vs 8.9 ± 3.5 months; *t* = −0.165, *P* = .869), as was prior kidney transplantation rotation time (2.7 ± 0.9 vs 2.5 ± 1.0 months; *t* = 1.128, *P* = .259). Baseline written examination scores were also comparable (72.0 ± 9.3 vs 71.8 ± 7.6; *t* = 0.126, *P* = .900). These findings indicate that the 2 groups were well balanced at baseline with respect to demographic characteristics, training exposure, and initial theoretical performance (Table 1).

**Table 1:** Baseline characteristics of trainees in the control and PBL groups.

Variable	Control group (n = 56)	PBL group (n = 59)	Test statistic	*P* value
Age (yr)	30.9 ± 2.8	31.4 ± 2.4	*t* = −1.026	0.305
Sex, male, n (%)	30 (53.6)	33 (55.9)	χ^2^ = 0.065	0.799
Training level, n (%)				
PGY-1	21 (37.5)	21 (35.6)		
PGY-2	20 (35.7)	16 (27.1)	χ^2^ = 1.692	.429
PGY-3	15 (26.8)	22 (37.3)		
Prior nephrology training (mo)	8.8 ± 3.0	8.9 ± 3.5	*t* = −0.165	.869
Prior kidney transplantation rotation (mo)	2.7 ± 0.9	2.5 ± 1.0	*t* = 1.128	.259
Baseline written examination score	72.0 ± 9.3	71.8 ± 7.6	*t* = 0.126	.900

PBL = problem-based learning, PGY = postgraduate year.

### 3.2. Theoretical knowledge assessment

Theoretical knowledge was evaluated using a standardized written examination covering donor and recipient evaluation, perioperative management, immunologic risk assessment, immunosuppressive drug monitoring, and early and late postoperative complications, with scores recorded on a 100-point scale and subdomains scored on a 0 to 20 scale. At the end of the training period, the PBL group achieved a significantly higher overall theoretical score than the control group (87.5 ± 5.9 vs 81.2 ± 6.8 points; *t* = −5.295, *P* < .001). Subdomain analysis showed that the PBL group performed better in donor and recipient evaluation (18.0 ± 1.6 vs 16.1 ± 2.0; *t* = −5.607, *P* < .001), perioperative management (17.9 ± 1.8 vs 16.3 ± 2.1; *t* = −4.376, *P* < .001), immunologic risk assessment (17.3 ± 1.9 vs 15.8 ± 2.3; *t* = −3.802, *P* < .001), and immunosuppressive drug monitoring (17.5 ± 1.8 vs 16.0 ± 2.2; *t* = −3.990, *P* < .001). Scores for early and late complication management were also higher in the PBL group (17.8 ± 1.7 vs 16.9 ± 2.4; *t* = −2.310, *P* = .023). In addition, the proportion of trainees achieving an overall exam score ≥ 80 points was significantly greater in the PBL group than in the control group (88.1% vs 64.3%; χ^2^ = 9.096, *P* = .003). These findings indicate that the PBL-based training protocol was associated with superior post-training theoretical performance across multiple key domains of kidney transplantation (Table 2).

**Table 2:** Theoretical knowledge performance of trainees in the control and PBL groups.

Variable	Control group (n = 56)	PBL group (n = 59)	Test statistic	*P* value
Overall post-training theoretical score (0–100)	81.2 ± 6.8	87.5 ± 5.9	*t* = −5.295	<.001
Donor and recipient evaluation (0–20)	16.1 ± 2.0	18.0 ± 1.6	*t* = −5.607	<.001
Perioperative management (0–20)	16.3 ± 2.1	17.9 ± 1.8	*t* = −4.376	<.001
Immunologic risk assessment (0–20)	15.8 ± 2.3	17.3 ± 1.9	*t* = −3.802	<.001
Immunosuppressive drug monitoring (0–20)	16.0 ± 2.2	17.5 ± 1.8	*t* = −3.990	<.001
Early and late complication management (0–20)	16.9 ± 2.4	17.8 ± 1.7	*t* = −2.310	.023
Overall score ≥ 80, n (%)	36 (64.3)	52 (88.1)	χ^2^ = 9.096	.003

PBL = problem-based learning.

### 3.3. Clinical reasoning ability and multidisciplinary case discussion performance

Clinical reasoning ability, assessed by structured case analysis, was higher in the PBL group than in the control group. The PBL group achieved a greater overall clinical reasoning score (85.0 ± 6.0 vs 78.0 ± 7.0; *t* = −5.744, *P* < .001). Subscale analysis showed that trainees in the PBL group obtained higher scores in problem identification (21.5 ± 2.0 vs 19.0 ± 2.5; *t* = −5.902, *P* < .001), diagnostic reasoning (21.8 ± 2.1 vs 19.5 ± 2.4; *t* = −5.458, *P* < .001), management strategy formulation (21.4 ± 2.0 vs 19.2 ± 2.6; *t* = −5.067, *P* < .001), and evidence justification (22.0 ± 2.0 vs 20.0 ± 2.3; *t* = −4.965, *P* < .001). The proportion of trainees achieving an overall clinical reasoning score ≥ 80 points was higher in the PBL group than in the control group (81.4% vs 53.6%; χ^2^ = 10.164, *P* = .001). Performance in multidisciplinary kidney transplantation case discussions showed a similar pattern. The overall discussion performance score was higher in the PBL group compared with the control group (86.0 ± 6.0 vs 79.0 ± 7.0; *t* = −5.744, *P* < .001). The PBL group also obtained higher scores in accuracy of clinical interpretation (22.0 ± 2.0 vs 19.8 ± 2.4; *t* = −5.326, *P* < .001), completeness of problem identification (21.4 ± 2.1 vs 19.0 ± 2.5; *t* = −5.560, *P* < .001), clarity of reasoning and presentation (21.6 ± 2.2 vs 19.3 ± 2.6; *t* = −5.108, *P* < .001), and integration of multidisciplinary information (22.2 ± 2.0 vs 20.0 ± 2.4; *t* = −5.326, *P* < .001). Consistently, a higher proportion of trainees in the PBL group had an overall discussion performance score ≥ 80 points compared with the control group (83.1% vs 55.4%; χ^2^ = 10.407, *P* = .001; Table 3).

**Table 3:** Clinical reasoning ability and multidisciplinary case discussion performance in the control and PBL groups.

Variable	Control group (n = 56)	PBL group (n = 59)	Test statistic	*P* value
Clinical reasoning ability				
Overall clinical reasoning score (0–100)	78.0 ± 7.0	85.0 ± 6.0	*t* = −5.744	<.001
Problem identification (0–25)	19.0 ± 2.5	21.5 ± 2.0	*t* = −5.902	<.001
Diagnostic reasoning (0–25)	19.5 ± 2.4	21.8 ± 2.1	*t* = −5.458	<.001
Management strategy formulation (0–25)	19.2 ± 2.6	21.4 ± 2.0	*t* = −5.067	<.001
Evidence justification (0–25)	20.0 ± 2.3	22.0 ± 2.0	*t* = −4.965	<.001
Overall clinical reasoning score ≥ 80, n (%)	30 (53.6)	48 (81.4)	χ^2^ = 10.164	.001
Multidisciplinary case discussion performance				
Overall discussion performance score (0–100)	79.0 ± 7.0	86.0 ± 6.0	*t* = −5.744	<.001
Accuracy of clinical interpretation (0–25)	19.8 ± 2.4	22.0 ± 2.0	*t* = −5.326	<.001
Completeness of problem identification (0–25)	19.0 ± 2.5	21.4 ± 2.1	*t* = −5.560	<.001
Clarity of reasoning and presentation (0–25)	19.3 ± 2.6	21.6 ± 2.2	*t* = −5.108	<.001
Integration of multidisciplinary information (0–25)	20.0 ± 2.4	22.2 ± 2.0	*t* = −5.326	<.001
Overall discussion score ≥ 80, n (%)	31 (55.4)	49 (83.1)	χ^2^ = 10.407	.001

PBL = problem-based learning.

### 3.4. OSCE performance

The overall OSCE score was higher in the PBL group than in the control group (85.3 ± 6.5 vs 78.5 ± 7.2 points; *t* = −5.307, *P* < .001). Analysis of individual stations showed that the PBL group achieved higher scores for postoperative monitoring (21.4 ± 2.1 vs 19.0 ± 2.6; *t* = −5.429, *P* < .001), interpretation of immunosuppressant drug levels (21.6 ± 2.0 vs 19.2 ± 2.5; *t* = −5.666, *P* < .001), recognition of acute rejection signs (21.5 ± 2.2 vs 19.1 ± 2.7; *t* = −5.210, *P* < .001), and communication in transplant counseling (21.7 ± 2.1 vs 19.3 ± 2.4; *t* = −5.695, *P* < .001). In addition, the proportion of trainees with an overall OSCE score ≥ 80 points was higher in the PBL group than in the control group (81.4% vs 51.8%; χ^2^ = 11.355, *P* = .001). These findings suggest that the PBL-based training protocol was associated with better performance in procedure-related skills and clinical application within the kidney transplantation OSCE (Table 4).

**Table 4:** OSCE performance of trainees in the control and PBL groups.

Variable	Control group (n = 56)	PBL group (n = 59)	Test statistic	*P* value
Overall OSCE score (0–100)	78.5 ± 7.2	85.3 ± 6.5	*t* = −5.307	<.001
Postoperative monitoring station (0–25)	19.0 ± 2.6	21.4 ± 2.1	*t* = −5.429	<.001
Interpretation of immunosuppressant drug levels (0–25)	19.2 ± 2.5	21.6 ± 2.0	*t* = −5.666	<.001
Recognition of acute rejection signs (0–25)	19.1 ± 2.7	21.5 ± 2.2	*t* = −5.210	<.001
Communication in transplant counseling (0–25)	19.3 ± 2.4	21.7 ± 2.1	*t* = −5.695	<.001
Overall OSCE score ≥ 80, n (%)	29 (51.8)	48 (81.4)	χ^2^ = 11.355	*P* = .001

OSCE = objective structured clinical examination, PBL = problem-based learning.

### 3.5. Trainee satisfaction with the teaching protocol

The PBL group reported higher overall satisfaction scores than the control group (4.4 ± 0.4 vs 3.9 ± 0.5; *t* = −5.902, *P* < .001). Compared with the control group, the PBL group showed higher satisfaction with teaching content (4.5 ± 0.4 vs 4.0 ± 0.5; *t* = −5.902, *P* < .001) and teaching format (4.4 ± 0.5 vs 3.8 ± 0.6; *t* = −5.810, *P* < .001). Perceived relevance of the training to clinical practice was also higher in the PBL group (4.5 ± 0.4 vs 3.9 ± 0.5; *t* = −7.083, *P* < .001), as was the overall learning experience score (4.4 ± 0.5 vs 3.9 ± 0.6; *t* = −4.841, *P* < .001). In addition, the proportion of trainees with an overall satisfaction score ≥ 4 points was significantly higher in the PBL group than in the control group (89.8% vs 64.3%; χ^2^ = 10.715, *P* = .001). These findings indicate that the PBL-based training protocol was associated with higher trainee satisfaction across multiple dimensions of the educational experience (Table 5).

**Table 5:** Satisfaction with the teaching protocol in the control and PBL groups.

Variable	Control group (n = 56)	PBL group (n = 59)	Test statistic	*P* value
Overall satisfaction score (1–5)	3.9 ± 0.5	4.4 ± 0.4	*t* = −5.902	<.001
Satisfaction with teaching content (1–5)	4.0 ± 0.5	4.5 ± 0.4	*t* = −5.902	<.001
Satisfaction with teaching format (1–5)	3.8 ± 0.6	4.4 ± 0.5	*t* = −5.810	<.001
Perceived relevance to clinical practice (1–5)	3.9 ± 0.5	4.5 ± 0.4	*t* = −7.083	<.001
Overall learning experience (1–5)	3.9 ± 0.6	4.4 ± 0.5	*t* = −4.841	<.001
Overall satisfaction score ≥ 4, n (%)	36 (64.3)	53 (89.8)	χ^2^ = 10.715	.001

PBL = problem-based learning.

## 4. Discussion

This study examined the application and effectiveness of a PBL-based training protocol in specialist physician education for kidney transplantation. The results showed that, compared with traditional teaching, PBL was associated with higher post-training theoretical examination scores, stronger clinical reasoning, better performance in multidisciplinary case discussions, superior OSCE outcomes, and greater trainee satisfaction. These findings suggest that PBL not only improves knowledge acquisition but also supports the development of higher-order cognitive and practical competences that are essential for complex transplant care.

The improvement in theoretical knowledge across all major domains of kidney transplantation indicates that PBL did not compromise the breadth of content coverage, despite its emphasis on case-based discussions and self-directed learning. The higher total examination scores and consistently better performance in donor–recipient evaluation, perioperative management, immunologic risk assessment, immunosuppressive drug monitoring, and complication management suggest that PBL may facilitate deeper understanding and more integrated knowledge structures. The requirement for trainees to formulate learning objectives from clinical problems, retrieve evidence, and apply it back to the case likely promoted organization and consolidation of key transplant concepts.^[8]^ This pattern is consistent with the theory that PBL enhances meaningful learning by linking new information to clinically relevant contexts rather than relying on rote memorization.

The observed advantages in clinical reasoning and multidisciplinary discussion performance further support the role of PBL in fostering complex cognitive skills. Higher scores in problem identification, diagnostic reasoning, management strategy formulation, and evidence justification suggest that PBL helped trainees structure clinical problems more systematically and select management plans on the basis of explicit reasoning rather than pattern recognition alone. Similarly, higher scores for accuracy of interpretation, completeness of problem identification, clarity of reasoning, and integration of multidisciplinary information in case discussions indicate that PBL may facilitate collaborative reasoning and communication within transplant teams.^[9,10]^ Given that kidney transplantation requires coordination among surgery, nephrology, immunology, and infectious diseases, the ability to synthesize information from multiple perspectives is central to safe and effective practice. The higher proportion of trainees achieving high-level performance (≥80 points) in both clinical reasoning and multidisciplinary discussions suggests that these benefits were not limited to a small subset of learners.

From an educational theory perspective, the observed benefits of PBL in this study can be interpreted through the lens of constructivist learning theory, which posits that learners actively construct knowledge through engagement with authentic problems rather than passively receiving information. In the context of kidney transplantation, where clinical decisions require integration of surgical, nephrological, immunological, and infectious disease knowledge, PBL provides a framework that aligns closely with real-world cognitive demands. Furthermore, situated learning theory emphasizes that learning is most effective when it occurs within the context in which it will be applied. The use of realistic transplant cases, multidisciplinary discussions, and scenario-based reasoning in the PBL curriculum may have facilitated the transfer of knowledge to clinical practice, as reflected by higher OSCE and discussion performance.

The OSCE findings demonstrate that the advantages of PBL extended beyond cognitive domains to observable performance in simulated clinical tasks. The PBL group achieved higher overall OSCE scores and better performance at individual stations assessing postoperative monitoring, interpretation of immunosuppressant drug levels, recognition of acute rejection, and communication in transplant counseling. These results imply that PBL may support transfer of knowledge and reasoning into practical behaviors, including procedural decision-making and clinician–patient communication. The alignment of PBL cases with real-world transplant scenarios, combined with repeated opportunities for feedback during tutorials and discussions, may have contributed to the consolidation of practical skills that are readily expressed in OSCE settings. Higher satisfaction scores in the PBL group across teaching content, teaching format, perceived relevance to clinical practice, and overall learning experience indicate that trainees regarded PBL as a more acceptable and engaging approach than traditional teaching.^[10,11]^ Perceived relevance to practice is particularly important in postgraduate specialist training, where adult learners often judge educational value based on its applicability to clinical work. The greater satisfaction may also reinforce learning behaviors, including preparation for tutorials, active participation, and sustained engagement with transplant-related literature.

The present findings are broadly consistent with recent evidence on the effectiveness of PBL in medical and health professions education. A recent global review highlighted that PBL-based curricula tend to integrate theoretical knowledge with clinical scenarios, thereby promoting active learning, critical thinking, and clinical reasoning across multiple educational levels.^[12]^ Several recent systematic reviews and meta-analyses have reported that PBL significantly improves critical thinking and problem-solving skills among medical and nursing students compared with traditional lecture-based instruction.^[1]^ These findings align with the present observation that PBL trainees achieved higher scores in clinical reasoning and evidence justification, suggesting that the mechanisms by which PBL enhances generic critical thinking may extend to specialist transplant training. Recent work in residency and advanced clinical training has also shown benefits of PBL and PBL-like small-group methods for clinical reasoning and self-directed learning. Hou et al reported that PBL integrated with AI-supported resources improved critical thinking and clinical reasoning in residency training, emphasizing active engagement with complex cases and iterative hypothesis testing.^[13]^ Similarly, Greenspan et al described how digital PBL platforms designed around diagnostic cases enhanced clinical reasoning processes through structured feedback and iterative decision-making.^[14]^ These observations are congruent with the higher scores in diagnostic reasoning and management strategy formulation in the PBL group in the current study, where learners were repeatedly required to interpret transplant-related cases and justify their choices.

In terms of practical skills, recent work has documented that PBL and active learning approaches may complement simulation-based and OSCE-based training by strengthening the cognitive framework that underpins procedural performance. Studies in surgical and procedural disciplines have shown that structured educational interventions combining case-based learning with simulation or OSCE assessments improve technical performance and clinical judgment in controlled settings.^[15]^ The present finding that PBL was associated with higher OSCE scores across stations related to monitoring, drug level interpretation, rejection recognition, and communication is consistent with this literature and suggests that PBL can be integrated with OSCE-based evaluation in transplant training. Although studies focusing specifically on physician education in kidney transplantation remain limited, there is increasing recognition of the need for structured educational programs in transplantation, including for patients and multidisciplinary teams. For example, Nilsson et al used a Delphi process to design internet-based education for patients awaiting kidney transplantation, highlighting the complexity of transplant-related information and the importance of tailored educational strategies.^[16]^ The present study extends this educational focus to specialist physicians by demonstrating that a structured PBL curriculum can enhance transplant-specific knowledge, reasoning, and skills. Taken together, these findings support the view that kidney transplantation, as a complex field with high stakes and multidisciplinary requirements, may particularly benefit from student-centered methods that require active engagement with realistic clinical problems.

From a clinical perspective, the findings suggest that implementing PBL in specialist physician training for kidney transplantation may contribute to more consistent and higher-level competence among trainees across cognitive, practical, and communication domains. Better performance in donor–recipient evaluation, immunologic risk assessment, immunosuppressive drug management, and recognition of rejection indicates that PBL-trained physicians may be better prepared to make timely and accurate decisions in real clinical settings. Although this study did not directly evaluate patient outcomes, the observed improvements in OSCE performance and multidisciplinary discussion suggest potential downstream benefits for perioperative safety, optimization of immunosuppressive regimens, and shared decision-making with patients and families. This study has several strengths. First, it evaluated multiple dimensions of educational outcomes, including theoretical knowledge, clinical reasoning, multidisciplinary case discussion, OSCE performance, and satisfaction, thereby providing a comprehensive picture of the impact of PBL on specialist transplant training. Second, the use of structured assessment tools, predefined scoring rubrics, and OSCE stations anchored in transplant-specific tasks increased the internal consistency of outcome measures. Third, baseline comparability between groups in demographic characteristics, prior training exposure, and initial theoretical performance reduced the likelihood that the observed differences were attributable to preexisting imbalances.

However, several limitations should be considered when interpreting the findings. The study was conducted at a single institution, which may limit generalizability to other settings with different curricula, case volumes, or trainee characteristics. The quasi-experimental design without randomization introduces the possibility of selection bias and unmeasured confounding, despite baseline similarity in observed variables. The outcomes were limited to educational measures and did not include longer-term indicators such as retention of knowledge, performance in independent clinical practice, or patient-level outcomes. Second, examiners for theoretical assessments, clinical reasoning evaluations, and OSCE stations were not blinded to group allocation. This may have introduced assessment bias, particularly for subjective components such as clinical reasoning and communication performance. In addition, the assessments of clinical reasoning and multidisciplinary discussion, although based on structured rubrics, may still be subject to rater bias. Finally, the study did not compare different intensities or formats of PBL, and therefore cannot determine the minimum effective “dose” or the relative contribution of specific PBL components. Future multicenter, prospective, and ideally randomized studies with longitudinal follow-up and linkage to clinical performance metrics are warranted to validate and extend these findings.

In addition, the implementation of a new PBL curriculum may have been associated with increased instructor enthusiasm, heightened trainee attention, and potentially greater allocation of educational resources, which could have contributed to improved outcomes independent of the instructional method itself (Hawthorne effect). These factors may limit the internal validity of the findings. Moreover, as this study was conducted in a single tertiary transplant center with specific institutional resources and faculty expertise, the generalizability of the results to other training environments may be limited.

## 5. Conclusion

This study demonstrated that a PBL-based instructional model significantly enhanced multiple core competencies required in kidney transplantation training. Compared with traditional teaching, PBL resulted in superior theoretical knowledge, stronger clinical reasoning, improved multidisciplinary discussion performance, and higher OSCE scores. Trainee satisfaction was consistently higher across all domains, suggesting broader educational acceptability. Collectively, these findings indicate that PBL represents an effective and comprehensive pedagogical approach for specialist physician training in kidney transplantation, although further prospective and multicenter studies are needed to confirm these observations.

## Author contributions

**Conceptualization:** Chen Gao, Jiyuan Li, Kankan Shui, Xubiao Xie.

**Data curation:** Chen Gao, Jiyuan Li, Kankan Shui, Xubiao Xie, Longkai Peng.

**Formal analysis:** Chen Gao, Jiyuan Li, Kankan Shui, Xubiao Xie.

**Funding acquisition:** Chen Gao.

**Investigation:** Chen Gao.

**Writing** – **original draft:** Chen Gao, Longkai Peng.

**Writing** – **review & editing:** Chen Gao, Longkai Peng.

## References

[1] Wei B Wang H Li F. Effectiveness of problem-based learning on development of nursing students’ critical thinking skills: a systematic review and meta-analysis. Nurse Educ. 2024;49:E115–9. 38016174 10.1097/NNE.0000000000001548

[2] Lim WK. Problem based learning in medical education: handling objections and sustainable implementation. Adv Med Educ Pract. 2023;14:1453–60. 38164409 10.2147/AMEP.S444566 PMC10758192

[3] Fyrenius A Bergdahl B Silén C. Lectures in problem-based learning--why, when and how? An example of interactive lecturing that stimulates meaningful learning. Med Teach. 2005;27:61–5. 16147772 10.1080/01421590400016365

[4] Mensour EA Tran C Li T Mallawaarachchi I Shaw JM Blissett S. Evaluating the outcomes of problem-based learning in postgraduate medical education: a systematic review and meta-analysis. Can Med Educ J. 2025;16:89–99. 10.36834/cmej.77394 PMC11931181 40135135

[5] Buckley S Coleman J Davison I. The educational effects of portfolios on undergraduate student learning: a best evidence medical education (BEME) systematic review. BEME Guide No. 11. Med Teach. 2009;31:282–98. 19404891 10.1080/01421590902889897

[6] Godoi A Koimtzis G Felix N. Educational interventions improve disparities in patient access to kidney transplantation: a network meta-analysis of randomized controlled trials. Int J Surg. 2024;110:8151–60. 39806752 10.1097/JS9.0000000000002154 PMC11634108

[7] Carrera LI Tellez TE D’Ottavio AE. Implementing a problem-based learning curriculum in an Argentinean medical school: implications for developing countries. Acad Med. 2003;78:798–801. 12915370 10.1097/00001888-200308000-00010

[8] Yeo S Chang BH. Implementation of problem-based learning in medical education in Korea. Korean J Med Educ. 2017;29:271–82. 29207458 10.3946/kjme.2017.73 PMC5717415

[9] Khoo HE. Implementation of problem-based learning in Asian medical schools and students’ perceptions of their experience. Med Educ. 2003;37:401–9. 12709180 10.1046/j.1365-2923.2003.01489.x

[10] Leonforte F Veroux P Mistretta A. Role of educational level in kidney transplant outcomes. Biomedicines. 2025;13:916. 40299545 10.3390/biomedicines13040916 PMC12024966

[11] Burgess A Bleasel J Haq I. Team-based learning (TBL) in the medical curriculum: better than PBL? BMC Med Educ. 2017;17:243. 29221459 10.1186/s12909-017-1068-z PMC5723088

[12] de Andrade Gomes J Braga LAM Cabral BP Lopes RM Mota FB. Problem-based learning in medical education: a global research landscape of the last ten years (2013-2022). Med Sci Educ. 2024;34:551–60. 38887406 10.1007/s40670-024-02003-1 PMC11180071

[13] Hou J An F Qin H. Application of DeepSeek-assisted problem-based learning in hematology residency training. BMC Med Educ. 2025;25:1291. 41039507 10.1186/s12909-025-07852-x PMC12492539

[14] Greenspan AA Goldberg GS Hamilton KL. Problem-based learning and digital platforms in medical education. Front Educ. 2025;10:1631337.

[15] Shahrezaei A Sohani M Taherkhani S Zarghami SY. The impact of surgical simulation and training technologies on general surgery education. BMC Med Educ. 2024;24:1297. 39538209 10.1186/s12909-024-06299-w PMC11558898

[16] Nilsson K Andersson G Johansson P Lundgren J. Developing and designing an internet-based support and education program for patients awaiting kidney transplantation with deceased donors through: a Delphi study. BMC Nephrol. 2023;24:311. 37880582 10.1186/s12882-023-03364-2 PMC10601218

